# Oxytocin Receptor Signaling in Vascular Function and Stroke

**DOI:** 10.3389/fnins.2020.574499

**Published:** 2020-09-25

**Authors:** Erin C. McKay, Scott E. Counts

**Affiliations:** ^1^Department of Translational Neuroscience, Michigan State University, Grand Rapids, MI, United States; ^2^Neuroscience Program, Michigan State University, East Lansing, MI, United States; ^3^Department of Family Medicine, Michigan State University, Grand Rapids, MI, United States; ^4^Hauenstein Neurosciences Center, Mercy Health Saint Mary’s Hospital, Grand Rapids, MI, United States; ^5^Michigan Alzheimer’s Disease Research Center, Ann Arbor, MI, United States

**Keywords:** oxytocin receptor, oxytocin, cerebrovasculature, cognition, stroke

## Abstract

The oxytocin receptor (OXTR) is a G protein-coupled receptor with a diverse repertoire of intracellular signaling pathways, which are activated in response to binding oxytocin (OXT) and a similar nonapeptide, vasopressin. This review summarizes the cell and molecular biology of the OXTR and its downstream signaling cascades, particularly focusing on the vasoactive functions of OXTR signaling in humans and animal models, as well as the clinical applications of OXTR targeting cerebrovascular accidents.

## Introduction

Since its original cloning and characterization by Kimura et al. (1992), to more recent predictions of its three dimensional structure (Busnelli et al., 2016), the human oxytocin receptor (OXTR) has garnered special attention for its role as a potential therapeutic target in a wide array of physiological and behavioral disorders. Several recent reviews have comprehensively covered the impact of OXTR signaling upon peripheral and central control of behavior and physiological functions including osmoregulatory, stress modulation, and memory (Jurek and Neumann, 2018; Grinevich and Neumann, 2020). By contrast, this review will survey the role of OXTR-mediated cellular and molecular pathways regulating vascular function, with a special focus on mechanisms of cerebrovascular disease and the receptor’s putative disease-modifying role in the post-stroke environment, which may be amenable to therapeutic targeting.

## The Oxytocin Receptor

The OXTR is a widely expressed G_αq_ protein-coupled receptor (GPCR) that binds its endogenous nonapeptide ligand, oxytocin (OXT), with an affinity of about 1–10 nM (Chini et al., 2017), as well as a structurally similar nonapeptide, vasopressin, with an affinity of about 100 nM–1 μM (Postina et al., 1996). The OXT peptide and its full nine amino acid sequence was first detailed in 1953 by Du Vigneaud and colleagues through varied partial hydrolysis experiments combined with paper chromatography (du Vigneaud et al., 1953). However, its existence was recognized as early as 1928 when researchers began testing the effects of OXT from pituitary extracts on peripheral reactions such as uterine contractions and blood pressure (Bourne and Burn, 1928; Griling and Eddy, 1928; Gruber, 1928; Kamm et al., 1928). OXT has since been found to exert both central and peripheral effects via OXTR-mediated phospholipase C (PLC) activation and downstream Ca^2+^ signal transduction (Zingg and Laporte, 2003). OXT is synthesized in the hypothalamic magnocellular and parvocellular neurons, reaching the peripheral circulation through the posterior pituitary (Argiolas and Gessa, 1991), while central actions appear to occur through both axonal and possibly volume transmission through dendrites (Meyer-Lindenberg et al., 2011). This volume transmission and its relative contribution to OXTR activation is an ongoing subject of debate, as more OXT neuronal projections to forebrain OXTR-expressing regions have become apparent in recent years (Knobloch et al., 2012; Grinevich et al., 2016). The very first hint of a bioactive OXTR was indirectly demonstrated by Sir Henry Dale and focused on the induction of uterine contractions by posterior pituitary gland components (Viero et al., 2010). Since that time OXTRs have not only been identified in the uterus (Fuchs et al., 1984), but also the mammary glands (Soloff et al., 1977), heart (Gutkowska et al., 1997), blood vessels (Thibonnier et al., 1999b), and brain (Muhlethaler et al., 1983). While the presence and activity of the receptor in each of these regions underscores the importance of OXTR signaling in peripheral and central physiology, this review will focus primarily on those found in the brain. Regardless, the widespread expression of this receptor and its ligands underscores the continued relevance and necessity of research into its functional repertoire (Grinevich et al., 2016), even after the 100 years that have passed since Dale engaged the receptor without knowing what it was (Viero et al., 2010). Before turning to physiological and disease modifying possibilities for the receptor, its biology as revealed by basic research will be summarized. This section provides a synthesis of the current understanding of the OXTR gene and protein at the cellular level that will be necessary to fully grasp the potential of its therapeutic use.

### The *Oxtr* Gene

In 1994, Inoue and colleagues described the genomic sequence of the human *Oxtr*, identifying it as a ∼17 kb single gene on chromosome 3 (Inoue et al., 1994). The gene contains two exons corresponding to the OXTR’s promotor region and two exons corresponding to the receptor coding sequence itself, along with three introns of which the third displays the longest sequence at 12 kb (Gimpl and Fahrenholz, 2001). Three transcripts of varying lengths were found in uterine tissues and differences were observed in antibody binding to the third intracellular loop (Adan, 1995), suggesting the existence of receptor subtypes at the time. However, it is now recognized that the three mRNA transcript lengths are due to sequence differences in the untranslated region (UTR) flanking a single coding sequence, thus resulting in one receptor transcript that may be differentially regulated post-transcriptionally (Breton et al., 1996; Gimpl and Fahrenholz, 2001). The *Oxtr* gene promoter also contains multiple response element sequences that contribute to differential expression of the receptor across age (Vaidyanathan and Hammock, 2017), region (Boccia et al., 2013), and at parturition in females (Insel, 1990; Young et al., 1997). The promotor region of the receptor has three TG-dinucleotide repeats that, based on the known ability of calcium to alter DNA structure at these repeats, could also explain some site-specific differences in OXTR mediated activity (Bale and Dorsa, 1997; Bale et al., 2001). Using kinase inhibitors, it has been demonstrated that regional differences exist in the brain as to whether protein kinase A or protein kinase C leads to increased *Oxtr* gene transcription (Bale et al., 2001). The 5′-UTR and promoter region of the *Oxtr* also bears putative response elements to interleukin-6, acute-phase proteins, GATA-1, c-Myb, and activator proteins 1 and 2 (Inoue et al., 1994). Beyond the promotor region, interest has also alighted on potential regulatory elements in the large third intronic region that separates the amino acid coding exons. Mizumoto et al. (1997) reported a region of hypomethylation in a central part of the intronic sequence in highly expressing myometrium and hypermethylation in low expressing leukocytes, suggesting epigenetic regulation of the receptor gene within the third intron. Furthermore, experience and exposure-induced epigenetic regulation of the expression of the *Oxtr* has been studied in relation to the emergence of characteristics of several conditions including autism spectrum disorders (ASD) and psychiatric conditions such as schizophrenia (Gregory et al., 2009; Bang et al., 2019; Andari et al., 2020).

### Protein Structure

**FIGURE 1:**
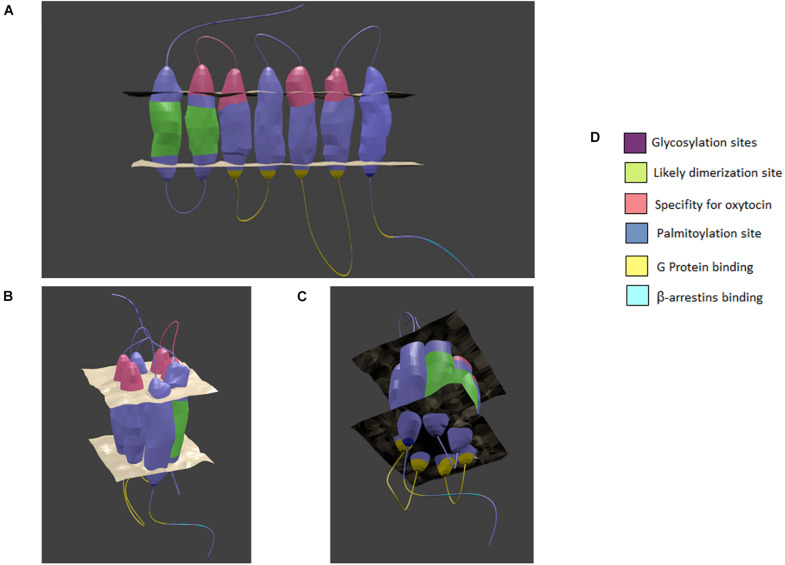
Model of the oxytocin receptor A. **(A)** linear view of the receptor. **(B)** The top of the receptor as it forms a binding pocket. **(C)** The bottom of the receptor as it forms a binding pocket. **(D)** Color coded key. The general location of specialized sites are highlighted by color with key provided. Based on chimera, point mutation, and structural analysis studies as cited in the text. Created in Blender 2.8.

Until recently, the tertiary structure of the OXTR had not been visualized with either X-ray crystallography or cryo-EM but rather by computer simulations, perhaps owing to its conserved homology with the rhodopsin family GPCRs in general and the beta-adrenergic receptors in particular (Fanelli et al., 1999). Indeed, many studies have pointed to it being a prototypical Class A GPCR (Busnelli and Chini, 2018). In a potentially exciting development, a crystal structure obtained by Waltenspühl and colleagues was recently reported that supports a canonical GPCR topology (https://doi.org/10.1101/2020.02.21.958090). This supports the rigor of prior structural studies of the OXTR, especially in the context of β-arrestin recruitment (Zhou et al., 2017) and dimerization (Busnelli et al., 2016), which have focused on comparisons to rhodopsin (Fanelli et al., 1999). The OXTR is composed of seven membrane-spanning α-helices with three intracellular and extracellular loops, a N-terminal region in the extracellular space, and a C-terminus in the cytoplasmic space (Barberis et al., 1998; Figure 1). Important individual structural elements of the OXTR, such as ligand binding sites, the site of interaction with the G_αq_ protein, transmembrane movements due to activation, and protein modifications have been revealed by computer simulation and mutation studies (Chini et al., 1995; Postina et al., 1996; Kimura et al., 1997; Barberis et al., 1998; Zhong et al., 2004; Busnelli et al., 2016; Zhou et al., 2017).

The OXTR binds both nonapeptides OXT and vasopressin, with OXT acting as a full agonist and vasopressin acting as a partial agonist (Chini et al., 1996). Of most relevance to this review is the similarity of vasopressin V1a receptors to OXTRs in structure, the G proteins and kinases to which they couple, and the high concentration of these receptors in the CNS and vasculature (Zingg, 1996). The peptides are predicted to assume a similar shape at physiological pH, consisting of a six-residue ring with a three-residue linear region (Barberis et al., 1998). The binding pocket for the ligands most likely lies in a cleft surrounded by the transmembrane domains. Chini and colleagues used a coordinated method of ligand and receptor mutations to find residues on the first extracellular loop of the receptor and transmembrane domains V and VI that accounted for the specificity of the receptor for OXT versus vasopressin (Mouillac et al., 1995; Chini et al., 1996). This occurs through aromatic-aromatic and hydrophobic-aromatic interactions with the isoleucine (I3) and leucine (L8) residues of OXT, which are the only two amino acids that differ between the two neuropeptides (Barberis et al., 1998). Therefore, any disruption in these residues would be expected to reduce the ability of the OXTR to respond to its natural agonist. The increase in availability of nonpeptide agonists and antagonists continue to contribute to the molecular modeling of this pore-based docking site, showing that its surface region promotes agonism and that deeper binding impairs conformational changes promoting antagonism (Uba et al., 2020). More broadly, the linear region of the peptide appears to closely interact with the first extracellular loop, and the cyclic region of OXTR appears to interact with the second extracellular loop with intermittent connectivity with transmembrane domains II-VII (Postina et al., 1996). A conserved aspartic acid residue in the second transmembrane domain (Asp 85) is demonstrably important for trafficking the OXTR to the plasma membrane (Schiffmann and Gimpl, 2018).

On the intracellular receptor face, research has focused on identifying the sites of G_αq_ protein subunit interactions, as well as the site of β-arrestin recruitment for receptor internalization. Selective replacement of the intracellular loops shows that intracellular loops 2 and 3 are vital for the ability of the receptor to effect G protein signaling, since no increase in phospholipase C is observed in their absence (Barberis et al., 1998). Furthermore, disruption of the large α-helix within the fourth intracellular domain also subsequently disturbs the ability of the receptor to bind G_αq_ (Zhong et al., 2004). V1aRs also couple to Gq/11 proteins and stimulate calcium-dependent signaling cascades (Zingg, 1996). The use of a X-ray free electron laser on crystalized rhodopsin in complex with arrestin revealed that phosphorylation at certain residues are vital to recruitment (Zhou et al., 2017). Briefly, the intracellular C-terminus of the receptor is phosphorylated at a β-sheet that attracts the N-terminus of arrestin, which allows it to undergo a conformational change or domain twist (Zhou et al., 2017). As such, a similar mechanism is likely to be involved in OXTR internalization and regulation of signaling activity. Additional OXTR posttranslational modifications include three N-glycosylation sites at residues N8, N15, and N26 in the N-terminus and sites for palmitoylation at cysteine residues C346 and C347 in the C-terminus; however, so far no vital role for these modifications have been defined (van Kesteren et al., 1996; Kimura et al., 1997; Barberis et al., 1998).

Recent GPCR research has revealed the ability of many GPCRs to dimerize or even oligomerize to maximize the strength of intracellular signaling (Cottet et al., 2010). Busnelli and colleagues recently demonstrated a likely presence of high affinity dimers of OXTR. Moreover, by using alkane spacers of varying lengths between ligands, they were able to identify a likely place of dimerization on the OXTR (Busnelli et al., 2016). The linkage probably occurs at the position of transmembrane helix 1 to transmembrane helix 2 which would accommodate bivalent ligands and allow for a reduction in entropy cost that would favor the formation of dimers (Busnelli et al., 2016). While methods need to advance before we can confirm the relative occurrence of single receptors, dimers, or oligomers, it provides an intriguing possibility for discrete manipulation of the OXT system, perhaps even for some of the conditions discussed below (Cottet et al., 2010). See Figure 1 for a visual summary.

### Signaling and Cellular Function

As noted above, activation of the OXTR typically stimulates intracellular Ca^2+^ mobilization through a PLC-dependent mechanism (Park et al., 1998; Gutkowska and Jankowski, 2012). While the OXTR is reported as coupling predominantly to G_αq/11_ type G protein subunits, it is now established that the OXTR also couples to G_i_/G_o_ type G protein complexes (Hoare et al., 1999; Busnelli and Chini, 2018). Recently, an ambitious synthesis of previous reports of OXTR intracellular signaling pathways was completed by Chatterjee and colleagues. To date, this open-source resource on NetPath is the most comprehensive overview of the OXTR signaling cascade (Chatterjee et al., 2016). Several of the more well-established pathways and their functional role at the cellular level will be described below. G_q/11_-transduced signaling is mediated by PLC-stimulated hydrolysis of the phospholipid phosphatidylinositol 4,5-bisphosphate (PIP_2_) to diacylglycerol (DAG), which in turns activates protein kinase C (PKC) and inositol 1,4,5-trisphosphate (IP_3_), which stimulates the release of intracellular Ca^2+^ stores via IP_3_ receptors and also activates PKC among other Ca^2+^-activated kinases (Thibonnier et al., 1999a; Viero et al., 2010).

Phospholipase C stimulates the phosphorylation of PI3K and AKT leading to the activation of endothelial nitric oxide synthase (eNOS) influencing cellular migration and vasodilation (Cattaneo et al., 2009; Viero et al., 2010). Additionally, the involvement of Rho kinases in smooth muscle uterine contractions suggests that OXTR activation of these kinases can lead to the production of phospholipase A2 and, in turn, cyclooxygenase 2 (Viero et al., 2010). The modulation of levels of Rho GTPases by OXTR leads to shifts in cell adhesion molecules, particularly in neurons (Zatkova et al., 2019). OXTR-stimulated PKC signaling has been shown to lead to the dephosphorylation of eukaryotic translocation factor eEF2, which aids in cellular proliferation through peptide chain elongation (Devost et al., 2008). Several other kinases are reported to be activated through the G_αq/11_ cascade of OXTR activation including the mitogen-activated protein kinases (MAPKs) ERK1/2, which induce c-fos and c-jun expression as an early mediator of proliferation, and ERK5, which is more specific to cellular differentiation (Zingg and Laporte, 2003; Devost et al., 2008). Whereas the suspected G_i_/G_o_ pathway is less well-defined, it has been demonstrated that G_iβγ_ signaling leads to p38 MAPK activation and aids cells in adaptive processes to physiological stressors through transcriptional activators and direct effects on cell stabilizing proteins (Hoare et al., 1999). Potential hyperpolarizing effects through G_i_ could occur through interactions with Ca^2+^-dependent K^+^ channels, as proposed based on recent studies using alternative peptides and chelators (Pierce et al., 2019). Hence, in addition to the well-established role for OXT as a contraction influencing hormone, it appears to be involved in cellular differentiation, migration, proliferation, responses to stressors, dilation, and inflammation.

**FIGURE 2:**
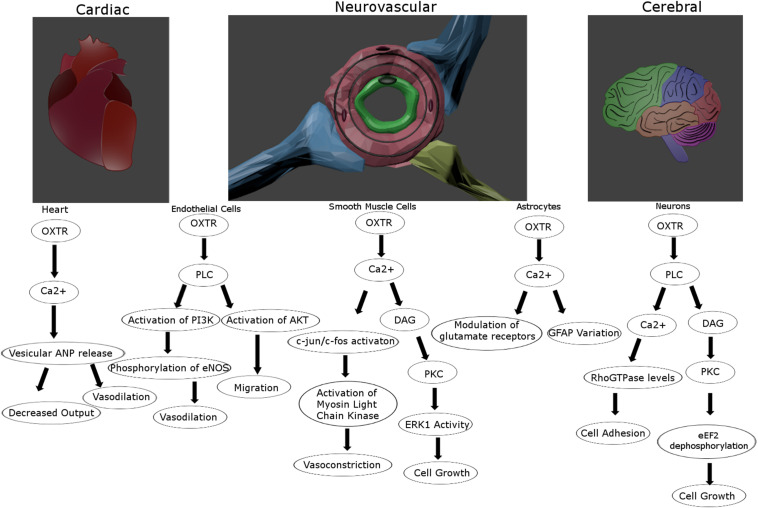
Proposed intracellular signaling pathways for the oxytocin receptor for vascular and cerebral cells. The oxytocin receptor is a vasoactive influencer and promotes proliferation and migration for multiple cell types in the tightly connected vascular and nervous systems. Created in Blender 2.8.

The response to OXTR signaling varies in a cell type-dependent manner (Figure 2). In smooth muscle cells and neurosecretory cells this results in contraction and excitability, respectively (Poulain and Wakerley, 1982; Soloff and Sweet, 1982). Importantly, smooth muscle cells respond to Ca^2+^ mobilization by triggering calmodulin to activate myosin-light chain kinase (Zingg and Laporte, 2003), most prominently at parturition leading to uterine contractions (Soloff et al., 1977). OXTR-secreting neurons of the hypothalamus express presynaptic OXTRs that are self-excitatory, which may impact the glutamatergic properties of subpopulations of these neurons (Hrabovszky and Liposits, 2008). Due at least partially to the neuromodulatory effect of OXT at OXTRs on neurons of multiple subtypes (Stoop, 2012), the general excitatory or inhibitory effects throughout the brain appears to differ and is further confounded by an influence on ionic channel expression (Bakos et al., 2018). For instance, it has excitatory effects at the hippocampal formation (Tiberiis et al., 1983), and presynaptic inhibitory effects in the SON (Hirasawa et al., 2001). Some studies have suggested an effect of OXTR activation on astrocytes including a modulation of glutamatergic signaling and GFAP induction that might be important for plasticity (Di Scala-Guenot and Strosser, 1992; Kuo et al., 2009; Wang and Hatton, 2009). Additional effects of OXTR signaling, whether stimulated by OXT or AVP, on CNS physiology and behavior will be discussed in greater detail below.

### Signaling Partners

As a receptor with a divergent, context-dependent signaling cascade, OXTR activity is influenced by interactions with a variety of additional cell and tissue-specific signaling partners and modulators. Many of these OXT and vasopressin binding-independent activities have involved a parallel expansion on the roles of known cellular components; among these are β-arrestins, cholesterol, and steroid hormones. Therefore, these connections have expanded not only our understanding of OXTR physiology, but also of GPCR-mediated cell biology.

Once thought to be only involved in the receptor desensitization process, the role of arrestins in receptor activation has recently been more fully elucidated. The β-arrestins have been suggested to promote alternative GPCR signaling pathways when ubiquitinated by sterically hindering interactions between the receptor and canonical pathway signaling partners and acting as a scaffold for enzymes driving alternative pathways (Shenoy and Lefkowitz, 2011). One such scaffolding activity that has been defined is the role of β-arrestin in recruiting cRAF-1, ERK2, and MEK1 to form the ERK2 signalosome, which can control nuclear transcription factors through phosphorylation (Luttrell et al., 2001). Another such β-arrestin role in signal transduction is its recruitment of the tyrosine kinase c-SRC for the purpose of mitogenic activity (Luttrell et al., 1999). While it is uncertain whether these pathways are also associated with the OXTR signaling repertoire, its known interactions with the β-arrestins suggest additional signal transduction properties initiated by internalized receptors.

Cholesterol is a lipoprotein long held to be an important building block of the plasma membrane, but GPCR research has also identified it as a potential factor in receptor activation. Work by Gimpl and colleagues identified a dual role for cholesterol in modulating OXTR signaling (Gimpl et al., 2002). When analyzing the role of varying cholesterol content on the binding of OXT to its receptor, they found a cooperative and facilitating role for cholesterol as an allosteric molecule for endogenous OXTR signaling (Gimpl et al., 2002). Specifically, chimeric approaches using the cholecystokinin type B receptor possessing a critical C-terminus region of the OXTR (amino acid residues C347-A389) suggest that the binding site for multiple molecules of cholesterol is located on the N-terminus of the receptor (Gimpl et al., 2002) or alternatively, due to specific residues on transmembrane domains 5 and 6 (Politowska et al., 2001). Further debate on the location of the cholesterol binding site may arise with the publication of the crystal structure of the OXTR, which suggests it is located within a pocket of transmembrane domains 4 and 5 (https://doi.org/10.1101/2020.02.21.958090). Secondarily, they reported that cholesterol protected OXTRs from degradation under high heat conditions (Gimpl and Fahrenholz, 2000). Further allosteric molecules include Mg^2+^, which is a positive allosteric molecule (Antoni and Chadio, 1989), and Na^2+^, which lowers the affinity of OXTR for its ligand in a concentration dependent manner (Schiffmann and Gimpl, 2018).

Estrogen may also play an essential role in OXTR signaling. In the uterus, the OXTR increases in density around parturition and decreases swiftly afterward (Soloff and Sweet, 1982). This increase is believed to be the consequence of a surge in the concentration of estrogen, with subsequent decreases due to progesterone (Soloff and Sweet, 1982). Further, predictable variances have also been noted around the estrous cycle in rats when estrogen and progesterone levels vary (Van Tol et al., 1988). Experiments using the protein synthesis inhibitor cycloheximide bolstered the belief that estrogen-induced OXTR expression is *de novo* synthesis-dependent and its decline in expression is due to progesterone’s antagonistic effect (Soloff and Sweet, 1982). In this regard, an estrogen response element (ERE) was found in the OXTR promoter region, and it has been reported that the addition of estrogen to the brain in pregnant rats resulted in increased OXTR expression in several brain regions (Young et al., 1997). Consistent with this, *in vitro* studies showed that cells transfected with the full palindromic ERE increased *Oxtr* promotor activity and increased protein levels, while a truncated ERE did not (Bale and Dorsa, 1997). Interesting, this effect of estrogen on *Oxtr* expression was not found in virgin dams, suggesting that additional factors associated with pregnancy synergize to upregulate the *Oxtr* gene (Young et al., 1997). While the complete story of steroid hormone influence on the OXTR remains to be elucidated, *in vivo* studies using mice deficient in estrogen receptor α (Young et al., 1998) suggest it is vital for OXTR increases due to estrogen. Studies of virally labeled OXTR in female ovariectomized rats with subsequent hormone replacement point to estrogens as potentially driving sexually dimorphic expression in certain regions where estrogen receptor α is co-expressed (Sharma et al., 2019). Also, *in vitro* binding studies suggest progesterone’s inhibition of OXTR binding density is due to a direct interaction with the OXTR (Grazzini et al., 1998); however, progesterone’s antagonistic effect toward estrogen also cannot be ignored (Soloff and Sweet, 1982).

### Cellular Processing

Once recruited from the endoplasmic reticulum to the cell surface (Dong et al., 2007), it is believed that usually the OXTR undergoes endocytosis and recycling upon stimulation (Conti et al., 2009). In order to trace the dynamic processing of the OXTR throughout the cell, we will focus on the process of receptor desensitization, internalization, trafficking, and recruitment.

Desensitization is a very important process to protect and preserve GPCR signaling as it both protects the cell from overstimulation, while also allowing for the recycling of receptors back to the cell surface for multiple responses over time, a discovery based on early studies of β-adrenergic receptors (Shear et al., 1976; Benovic et al., 1987; Smith et al., 2006). Varying time frames have been estimated for peak desensitization of the OXTR upon agonist stimulation. A range of 4–6 h for peak desensitization has been reported in myocytes expressing OXTR (Robinson et al., 2003), while others have reported it taking up to 20 h (Phaneuf et al., 1997). These differences can mostly be attributed to the concentration and tissue dependent aspects of receptor desensitization (Plested and Bernal, 2001).

Oxytocin receptor is considered a Class A GPCR with respect to its interaction with β-arrestins, meaning that it maintains a strong connection with β-arrestin to regulate its endocytosis into secretory or degradative pathways (Conti et al., 2009). The interaction of OXTR with β-arrestins, and therefore its desensitization, is initiated by phosphorylation of the receptor by G protein receptor kinase 6 (Grotegut et al., 2016, 2017). Studies on OXTR tagged with GFP suggest that many receptors are recycled back though the secretory pathway, indicating a more sustained need for OXTR pathway signaling. Indeed, fluorescence microscopy studies revealed the colocalization of fluorescently tagged OXTR with transferrin after stimulation and a subsequent return of the signal to the plasma membrane 4 h later independent of protein synthesis, suggesting prominent receptor recycling (Conti et al., 2009). Further detection of OXTR co-labeling with Rab5 and Rab4, small GTPases involved in “short cycle” trafficking back to the membrane, also support this conclusion (Conti et al., 2009). While this may be the typical cycle, this is not always the case, as β-arrestin-independent internalization and recycling loss has also been reported, particular with analogs of OXT (Passoni et al., 2016). Some nuclear trafficking of the OXTR in concert with β-arrestins, Rab5, importin-β, and transportin-1 has been reported in mouse osteoblasts, but any transcription modifying effects remain speculative (Di Benedetto et al., 2014).

Comparatively less is known about the trafficking of the receptor to the plasma membrane post-translation. Three N-linked glycosylation sites within the N-terminus of the OXTR suggest the importance of cell surface targeting of the receptor for cell function, although whether these sites are used for such a role is disputable (Kimura et al., 1997). As mentioned above, when the conserved residue Asp 85 is mutated in the OXTR, very little receptor makes it to the plasma membrane post-translation, suggesting a vital role for this amino acid in some stage of trafficking (Schiffmann and Gimpl, 2018). It is also unclear which Rab GTPases are involved in the initial trafficking from ER to Golgi to plasma membrane after translation (Dong et al., 2007). Given the established role the secretory pathway plays in controlling the number of receptors available at the cell surface, and therefore signaling magnitude (Dong et al., 2007), further research into OXTR trafficking dynamics will shed insights into its potential multifactorial role in cell and tissue function. Possible variances in desensitization and trafficking of the OXTR could have therapeutic implications based on drugs and individual differences, such as the divergence in pathways by different agonists (e.g., carbetocin vs. OXT) (Passoni et al., 2016). These variables may impact our understanding for OXTR-mediated functions with respect to vascular health, as discussed below.

## Oxtr Signaling in Vascular Health and Disease

### Vasoactive Agent

OXTR-regulated vasogenic activity has been well established from the earliest use of its peptide ligand in research (Gruber, 1928), when OXT was observed to lower blood pressure. This long-term decrease in blood pressure has subsequently been confirmed in rats (Petersson et al., 1996) and humans (Light et al., 2005). Subsequent studies in pregnant women and rats have shown that while blood pressure drops, heart rate increases with peripheral administration of OXT (Yashpal et al., 1987; Rabow et al., 2018). The interest in OXTR as a candidate in the etiology of and treatment for cardiovascular conditions has recently been revived due in large part to the work of Gutkowska and Jankowski (2012), which supports the concept of a protective effect for OXTR signaling in tissue response to infarctions that will be detailed below.

Oxytocin receptors are localized in the heart contributing to the release of atrial natriuretic peptide (ANP) and a decrease in cardiac output (Figure 2; Gutkowska et al., 1997). OXT can induce vasodilation when acting on endothelial cells through eNOS activation (Uvnäs-Moberg, 1998; Thibonnier et al., 1999b), but can also promote vasoconstriction when acting on smooth muscle cells (Altura and Altura, 1984; Figure 2). Further, there is reason to believe this might not be due to OXTR signaling, but through OXT acting on vasopressin receptors (Suzuki et al., 1992; Oyama et al., 1993). These divergent findings might also be vessel-dependent, as small artery vasodilation upon OXT administration has been reported (Rabow et al., 2018), while larger peripheral arteries may respond instead with vasoconstriction (Petersson, 2002). As Petersson points out, this could be explained by the alternate effects of OXTR activation upon endothelial versus smooth muscle cells, their relative distribution in large and small vessels, and the administration method (Petersson, 2002). OXTRs in the cerebrovasculature seem to maintain many of the same attributes as central receptors such as upregulation following circulating estrogen (Gutkowska et al., 2000), and interactions with arrestins and the same G proteins (Gutkowska and Jankowski, 2012). However, the blood pressure lowering effects of OXT seems to be based on receptors in the periphery and not those in the CNS, as peripherally but not centrally administered OXT lowers blood pressure (Petersson et al., 1998). A systemic rise in OXT concentration, even with intranasal delivery, leads to a decrease in regional cerebral blood flow, primarily in the amygdala (Martins et al., 2020). Alternatively, this same route of delivery under an fMRI using cerebral blood volume reported an increase in the hippocampus and frontal cortex (Galbusera et al., 2017). Although vasopressin can also activate OXTR receptors at high concentrations, vasoconstriction and increased heart rate via central administration of vasopressin is thought to be mediated by V1a receptors (Ohlstein and Berkowitz, 1986; Faraci, 1989; Loziæ et al., 2018). Hence, the cross reactivity between these ligands and receptors do seem to be independently distinguishable, and OXTR signaling clearly influences vascular activity in a context-dependent manner.

### Oxidative and Inflammatory Stress

Oxidative stress and inflammation are common to many neurodegenerating conditions, including those induced by an ischemic injury (Shukla et al., 2011; Chitnis and Weiner, 2017). An increase in antioxidant enzymes, activation of reactive oxygen species (ROS) producing enzymes, or decreased ROS directly are taken as evidence of a potential protective effect (Weber et al., 2008; Kahles and Brandes, 2012). Levels of pro- and anti-inflammatory cytokines as well as upstream activators are a means of identifying if there is evidence for inflammatory modulation (Barone and Parsons, 2000; Li X. et al., 2017). There is evidence from *in vitro* and *in vivo* studies that signaling through OXTR influences antioxidant and anti-inflammatory outcomes.

I*n vitro* studies have shown that exogenously applied OXT decreases the production of reactive oxygen species (ROS) initiated by H_2_O_2_ application to lymphocytes (Staniæ et al., 2016). This observation is supported by *in vivo* studies finding reduced ROS production with chronic OXT treatment in a mouse model of ASD (Wang et al., 2018) as well as reduced oxidative stress status in OXT-treated naïve Wistar rats (Honceriu et al., 2016), ischemia-reperfused Sprague-Dawley rats (Faghihi et al., 2012), and naïve zebrafish (Balmus et al., 2017). Candidate downstream enzymes mediating OXT’s effects on ROS production include MAPK/ERK1/2, superoxide dismutase (SOD), and glutathione as antioxidant promoting pathways (Deing et al., 2013; Polshekan et al., 2016; Wang et al., 2018). Moreover, NADPH oxidase-mediated production of ROS is observed to be dampened with the addition of OXT, indicating an effect of attenuating prooxidative pathways (Szeto et al., 2008; Rashed et al., 2011). Alternatively, OXTR knockdown in fibroblasts led to a decrease in oxidative stress and an increase in antioxidative enzymes (Deing et al., 2013). Critically, in a parallel but inverse set of discoveries, the use of OXTR antagonists lead to an increase in markers of oxidative stress in cardiac tissue (Simsek et al., 2012). In cardiac cells and *in vivo* rat hearts a protective effect for vasopressin, and specifically vasopressin acting at V1aR and OXTRs has been reported to reduce oxidative stress (Nazari et al., 2015; Ghorbanzadeh et al., 2020). So in regard to OXT and OXTR roles in oxidative stress, complete independence from the arginine-vasopressin system cannot be assumed. While the bulk of the evidence available points to a decrease in ROS following OXT administration or OXTR engagement, it is unclear whether encouraging antioxidative signals is the primary cause or a reduction in prooxidative ones, and whether enzymatic subtypes, cell type, or injury state matter.

With respect to the role of OXTR signaling in inflammatory modulation, complementary *in vitro* and *in vivo* studies have shown that OXT administration reduces the production of pro-inflammatory cytokines such as IL-6, TNF-α, and IL-1β (Szeto et al., 2008; Jankowski et al., 2010; Garrido-Urbani et al., 2018), and increases anti-inflammatory cytokines such as IL-10 and TGF-β (Jankowski et al., 2010; An et al., 2019). This dampening of proinflammatory cytokines is at least partially attributed to actions at NF-kβ (Yuan et al., 2016). Alternatively, stimulating the OXTR leads to a several fold reduction in the receptor for advanced glycation end-products (RAGE), which stimulates macrophage cells to produce proinflammatory cytokines (Metz et al., 2012). Additionally, cytokines like IL-6 and IL-1β appear to feed forward and increase the expression of OXTR (Young et al., 1997; Schmid et al., 2001), suggesting a protective feed-back loop. While less studied than OXT, the role of vasopressin in inflammation is highly variant in either enhancing or dampening inflammatory responses and this could be due to multiple receptor subtypes (Ameli et al., 2014; Jan et al., 2017; Xu et al., 2017). However, as far as specific inflammatory cell types, OXT seems to generally promote the activation of peripheral immune responses while tempering central immune activation (Li T. et al., 2017; Stary et al., 2019). For instance, OXT increases the production of spleen leukocytes, enhances the differentiation of thymus immune cells (Hansenne et al., 2005; Stary et al., 2019), and reduces inflammation-related transendothelial cell migration (Erkanli et al., 2013; Liu et al., 2017), whereas OXT appears to mitigate microglial activation in the brain (Yuan et al., 2016; Inoue et al., 2019; Mairesse et al., 2019). The elucidation of the full extent of OXTR signaling involved immune activation and the consequences of this activation on OXTR activity is an ongoing subject of research.

### The OXTR in Brain: Cerebrovascular Function and Post-Stroke Potential

The potential role for OXTR signaling in cerebrovascular protection is grounded in evidence for its protective role in the periphery. With respect to cardiovascular disease, OXTR stimulation in the heart causes the release of ANP and decreased heart rate (Gutkowska et al., 1997). In addition, a decrease in pressure in the chambers of OXT-treated hearts has also been reported (Costa-E-Sousa et al., 2005). Stress-induced increases in blood pressure that can prove deleterious over time are also mitigated with higher plasma OXT in mothers (Grewen and Light, 2011). Since ischemic heart disease is a leading cause of death worldwide (Naghavi et al., 2015), the idea that OXTR-mediated effects might prove effective as a management strategy or as an acute rescue agent has gained traction (Nilsson, 2009). OXT treatment in rodents has been shown to induce stem cells to adopt a cardiomyocyte phenotype (Matsuura et al., 2004), which could lead to exciting prospects in cardiac regeneration. Some experiments have even tested non-invasive ways to increase OXT after heart surgery, including massage and music interventions (Nilsson, 2009). In addition to these acute treatments, long term OXT increases are thought to be a primary agent through which social ties reduce the risk for cardiovascular disease (Knox and Uvnäs-Moberg, 1998). In rats, treatment with OXT lowered blood pressure in a hypertensive strain (Petersson and Uvnäs-Moberg, 2008), and prevented the occurrence of hypertension subsequent to hypoxic injury (Jameson et al., 2016), whereas hypertension induced by angiotensin-II was found to be exacerbated by OXT administration (Phie et al., 2015). In regards to cardiac sympathetic tone, one study examining myocardial infarction found a negative effect of OXT based increased sympathetic tone (Roy et al., 2018), while another examining ventricular hypertrophy and subsequent heart failure found a beneficial effect to this same OXT linked sympathetic tone based modulation (Garrott et al., 2017). This is an interesting paradox that could perhaps suggest a strong environmental effect to the effects of OXT. It could be that the response to OXT in an ischemic environment versus one of pathological remodeling could differ substantially. Regardless, well-controlled longitudinal studies are needed to assess whether OXTR manipulation might lead to improved cardiovascular or even cerebrovascular outcomes.

Oxytocin receptors might be uniquely positioned to respond to vascular insults due to their localization on microvascular endothelial cells (Thibonnier et al., 1999b; Nakamura et al., 2000). OXT has been shown to induce proliferation of endothelial cells, most likely through a PI3K and Src kinase dependent production of nitric oxide by eNOS (Cassoni, 2006; Cattaneo et al., 2008). Beyond these pro-angiogenic effects, the receptor appears to have potent anti-inflammatory and antioxidant properties. It both reduces the activity of NADPH oxidase isoforms on endothelial cells and innate immune cells (Szeto et al., 2008; Rashed et al., 2011) and reduces the production of pro-inflammatory cytokines in favor of anti-inflammatory cytokines (Jankowski et al., 2010; Wang et al., 2018). OXTRs also potentiate the uptake of glucose during hypoxia (Lee et al., 2008; Florian et al., 2010).

Notably, these are some of the same pathways that are thought to be beneficial in the recovery of surviving tissue after an ischemic injury (Ooboshi et al., 2005; Bir et al., 2012; Liu et al., 2013; Rodrigo et al., 2013; Choi et al., 2015). OXTR activation has been mechanistically linked to the amelioration of tissue damage following cardiac infarction (Jankowski et al., 2010), renal infarction (Tuǧtepe et al., 2007), hepatic infarction (Düşünceli et al., 2008), and cerebral stroke (Karelina et al., 2012; Moghadam et al., 2018; Seo et al., 2018). Cardiomyocytes can also be protected from ischemia and reperfusion injury through a reduction in mitochondrial-sourced ROS and a shift in cell signaling away from pro-apoptotic Bax toward anti-apoptotic Bcl-2 (Gonzalez-Reyes et al., 2015). In examining cerebral ischemic stroke more directly, Karelina and colleagues (Karelina et al., 2012) used social housing, OXT treatment, and OXTR antagonists to demonstrate a protective role for OXT in reducing tissue loss and deleterious inflammation while enhancing antioxidative enzyme expression following middle cerebral artery occlusion. This observation has been extended to show that the neuroprotective effect of nursing in cerebral ischemia can be mimicked with exogenous OXT administration in mice, reducing ROS production and apoptotic neuron death (Moghadam et al., 2018; Stary et al., 2019). Effects on cognitive changes post-stroke in animal models and human studies are limited. Only one human case study post-stroke has been published, wherein the authors speculated that a patient’s rapid recovery from post-partum stroke may have been due to OXT administered to reduce postpartum bleeding and increased endogenous OXT release upon contact with her newborn (Seo et al., 2018). Cognitive effects have been limited to post-stroke depression and anxiety-like behavior in animals, and supposition in humans (Long and Hillis, 2016; Zhong et al., 2020). Post-stroke memory impairments are a relatively unexplored target.

In this regard, a *de novo* up-regulation of OXTRs in astroglia within the peri-infarct space was demonstrated in patients who died with a clinical pathologic diagnosis of vascular dementia, suggesting a druggable target for quick intervention (McKay et al., 2019). This is supportive of the detection of functional OXTR on astroglial cells in culture that can bind appropriate radioligands and trigger a release of TGF-β (Di Scala-Guenot and Strosser, 1992; Mittaud et al., 2002), as well as reports of post-ischemic increases in OXTR for CNS tissue (Moghadam et al., 2018), though the opposite has been found in post-ischemic heart tissue (Jankowski et al., 2010). In cases of birth-related ischemic injury, OXT administration improved viability of immature hippocampal cells and reduced markers of oxidative stress (Tyzio et al., 2006; Ceanga et al., 2010; Kaneko et al., 2016), which may be linked to associated changes in GABAergic chlorine channels in addition to possible hemodynamic alterations (Tyzio et al., 2006; Kaneko et al., 2016). By contrast, other studies have found that OXT administered to dams of pups undergoing birth-related ischemic injury might actually exacerbate injury due to a vasodilatory reaction leading to exacerbated birth anoxia (Boksa et al., 2015). Critically, an ischemic environment might switch the vasodilatory effect of OXT to a vasoconstrictive one based on studies of isolated cerebral arterioles (Bari et al., 1997). In the case of long term management of vascular health, OXT has been found to reduce atherosclerosis in mice, rabbits and rats prone to the development of such plaques (Nation et al., 2010; Ahmed and Elosaily, 2011; Szeto et al., 2013). Interestingly, vasopressin might be protective against ischemic injury (Nazari et al., 2015), but V1aRs are thought to be deleterious and their antagonism may present a route of intervention (Ameli et al., 2014). While this work suggests that the OXTR is a valid target for recovery of cerebrovascular insults, including stroke related to cognitive impairment and dementia (McKay et al., 2019), further mechanistic and validation studies are warranted, especially in reference to ischemic conditions.

### Cell Survival

Another current strategy to improve outcomes post-ischemia is the enhancement of cell survival through inhibiting apoptosis (Caglayan et al., 2019; Chen et al., 2020; Jia et al., 2020), reducing excitotoxicity (Maiolino et al., 2019; Jia et al., 2020), and improving metabolism (Ren et al., 2008). The general measures of reduced apoptosis are taken as a reduction in caspase activation and a higher ratio of Bcl-2 to Bax (Chen et al., 2020; Jia et al., 2020). Combatting excitotoxicity often ultimately focuses either on reducing glutamate signaling, mostly through NMDA receptors, or reducing intracellular calcium accumulation and waves in connected cells (Maiolino et al., 2019; Pietrogrande et al., 2019). The improvement of metabolism is closely tied to glucose uptake to sustain cells in the absence of production (Ren et al., 2008). While current methods to enhance cell survival through these means include pharmacological intervention, pre-conditioning, and non-invasive neuronal stimulation, there is reason to believe that enhancement of cell survival in post-ischemic environment could also be enhanced through targeting of the OXTR as evidenced by overlap with the survival mechanisms targeted in these studies. Some of these mechanisms include the favoring of anti-apoptotic proteins over pro-apoptotic ones, potential suppression of NMDA receptor activation, and the enhancement of glucose uptake in some cell types.

Oxytocin treatment increases the expression of the pro-survival Bcl-2 in cases of ischemia/reperfusion injury, at least in cardiac tissue (Kobayashi et al., 2009; Alizadeh et al., 2012). A reduction in the pro-apoptotic Caspase-3 and Bax also supports the anti-apoptotic function of OXTR signaling (Erkanli Senturk et al., 2013; Erbas et al., 2017). Oxytocin administration dampens the basal levels of glutamatergic excitatory activity in the frontal cortex of mice, and the use of inhibitors suggests this occurs at the NMDA receptors (Ninan, 2011). Lastly, OXT administration enhances the uptake of glucose both peripherally in skeletal and cardiac cells (Lee et al., 2008; Florian et al., 2010), and centrally in non-human primates after intranasal administration (Arias del Razo et al., 2020). Another option is, again, the observed pro-survival and glucose metabolism supporting effects as evidenced by vasopressin administration and V1aR knockout animals (Aoyagi et al., 2007; Ghorbanzadeh et al., 2020). While the existing evidence is minimal, and little has been tested centrally, there are reports of some enhancement of cell survival, including under ischemic conditions, for OXT and OXTR signaling.

### Synaptic Plasticity and Neurogenesis

When it comes to CNS repair after injury, enhancing plasticity for the strengthening of remaining connections and neurogenesis for recovery are strong areas of focus (Onodera et al., 1990; Ohira et al., 2010; Nazari et al., 2016; Kisoh et al., 2017). The support of plasticity is assessed through the activation of kinases involved in the induction of long-term potentiation (LTP) and the modulation of synaptic receptors. Of the kinases involved in LTP studied in ischemic stroke the mitogen-activated protein kinases (MAPKs) are often cited (Davis and Vanhoutte, 2000; Komiyama et al., 2002), and the synaptic receptors like GABA_A_Rs (Clarkson et al., 2010; Kim et al., 2014). Neurogenesis in stroke studies is assessed both directly through markers like BrdU (Bartley et al., 2005), or through the production of growth factors (Naylor et al., 2005). Studies of OXT and the OXTR signaling pathways have revealed effects on plasticity and neurogenesis both in and outside of ischemic conditions. By contrast, vasopressin exerts little effect on neurogenesis outside of early development (Leuner et al., 2012), but an enhancement of plasticity in hippocampal subfields has been reported (Wang et al., 2001; Pagani et al., 2015).

Signaling cascades through the OXTR have been associated strongly with MAPKs, particularly ERK1/2 and ERK5 (Devost et al., 2008). The signaling of OXTR through these mechanisms are strongly associated with cellular proliferation (Tom and Assinder, 2010). Importantly, in hippocampal fields, a common site for the investigation of LTP, a facilitation of LTP by OXTR has been demonstrated to be dependent on MAPKs (Tomizawa et al., 2003; Lee et al., 2015). An important caveat to consider is the potential divergent effects of these signaling partners based on the cellular localization of receptors, namely whether they are present in caveolin domains or not (Guzzi et al., 2002; Rimoldi et al., 2003). From the time of its role in the GABAergic switch to an inhibitory one around birth, OXT is tied to GABAergic modulation (Tyzio et al., 2006). Now there is increasing interest in the OXT mediation of GABA_A_R signaling, particularly under ischemic stress (Kaneko et al., 2016). While the protective effects observed have been attributed to a counter to excitotoxicity (Kaneko et al., 2016), the role of GABA_A_Rs, especially reducing tonic inhibition of these receptors, in enhancing neuroplasticity is an intriguing alternative (Clarkson et al., 2010).

In regards to neurogenesis, OXTR positive neurons in the CA2 and CA3 subfields of the hippocampus undergo neurogenesis as detected by BrdU upon OXT stimulation, while the deletion of the receptor impairs survival (Lin et al., 2017). A similar effect is found in the hypothalamus, while an opposite effect is observed in the olfactory bulb upon OXT stimulation (Lévy et al., 2019). Differences in effects within regions of the dentate gyrus are also observed as well (Leuner et al., 2012). This suggests that the role of OXT and OXTR signaling in neurogenesis could be context dependent. Two potential effectors for this neurogenesis through OXTR signaling could be through Akt/PI3K signaling, which is tied to neurogenesis (Bruel-Jungerman et al., 2009; Zhang et al., 2009) and or through support of growth factors cited as determinants of neurogenesis and angiogenesis (Dempsey and Kalluri, 2007). One avenue of OXTR signaling is through phosphorylation and activation of Akt/PI3K (Gonzalez-Reyes et al., 2015). Several growth factors appear to be upregulated upon OXT stimulation such as brain-derived neurotrophic factor and insulin-like growth factor (Sirotkin et al., 1996; Havranek et al., 2015). In turn, OXT and OXTR expression is regulated by growth factors, especially insulin-like growth factor 1 (Holtorf et al., 1989). While the interactions are complex and likely conditionally dependent there is reason to investigate OXTR signaling as an influencer of neurogenesis and plasticity.

## The OXTR as a Therapeutic Target for Ischemic Injury

Oxytocin receptor signaling has long been exploited for therapeutic purposes, such as for inducing labor or halting preterm contractions, but its potential clinical applications might go far beyond that. For example, as discussed above, there might be CNS applications for OXTR signaling in ameliorating CVD related tissue loss, enhancing repair, and/or protecting cognition. However, this goal is hindered by the inability of current agonists and antagonists to cross the blood brain barrier (BBB) and the lack of specific compounds that do not also target vasopressin receptors. Despite these hindrances, the history of successfully targeting GPCRs, which represent the majority of drug targets (Sriram and Insel, 2018), and the well-established safety profile of OXT (Anagnostou et al., 2014; DeMayo et al., 2017). suggests that there is translational potential in targeting the OXTR for vascular disease.

As a GPCR, the OXTR is a member of a class of the most widely utilized therapeutic targets, as 35% of drugs on the market target GPCRs (Sriram and Insel, 2018). On the other hand, several hindrances have emerged that have slowed clinical and basic research aimed at targeting the OXTR. For example, endogenous OXT does not cross the BBB in large amounts, making central actions of the receptor difficult to manipulate (Mens et al., 1983). Additionally, many OXTR agonists or antagonists are not specific as they also show cross-reactivity with vasopressin receptors (Manning et al., 2012). Finally, disparate findings over the relative efficacy of peptide vs. non-peptide agonists for OXTR targeting have added a layer of complexity to development of novel therapeutic strategies (Evans et al., 1992; Freidinger and Pettibone, 1997; Manning et al., 2012). These challenges need to be overcome to truly test the extent to which OXTR signaling might modify the progression of cerebrovascular lesion spread, possible cognitive dysfunction, and/or additional functional impairments.

The BBB is relatively impenetrable except for gaseous and small molecules, especially to larger peptides or transmitters, though transporters exist to allow the gated passage of many other substances such as nutrients (Abbott et al., 2006; Daneman and Prat, 2015). Some studies have suggested that labeled OXT does cross the BBB, but comparisons of methods and results reveal that about 1 in 10,000 units of peripherally administered OXT reaches the CNS (Mens et al., 1983; Hollander et al., 2003). A recent finding by Yamamoto and colleagues that RAGE on vascular endothelial cells is the main transporter responsible for this CNS bioavailability presents an enticing possible means to enhance this penetrance (Yamamoto et al., 2019). Though, the issues with targeting such a pro-inflammatory receptor as RAGE raises multiple troubling caveats (Wautier et al., 2017). Similar problems arise with the administration of intranasal OXT, as some studies have found beneficial effects when the peptide is administered in this way (Alvares et al., 2010; Goodin et al., 2014), but again, CNS penetration is relatively low (Leng and Ludwig, 2016). For a hormone with known peripheral and central properties, delivering supraphysiological doses to the periphery to ensure central activation likely results in off-target effects. Perhaps this concern is not such a hindrance, as MacDonald and colleagues found a serviceable safety profile with the use of up to 40 IU OXT (MacDonald et al., 2011). The reported side effects were extremely rare, with the only severe side effect being water intoxication reported twice among over 1500 cases (MacDonald et al., 2011). A promising alternative is the use of aerosolized OXT that, like intranasal oxytocin, relies on the nasal epithelium for absorbance, but can cover relatively more of the surface (Modi et al., 2014; Simpson et al., 2017). Significantly, several studies have reported its ability to not only increase plasma OXT levels, but sustained increases in CSF OXT levels, as well (Modi et al., 2014; Simpson et al., 2017). Some suggestion has been made of using ultrasonic disruption of the BBB by focused pulses. While rodent and non-human primate studies have found no long-term negative consequences, the delivery of OXT or an analog has not been tested and the chance of hemorrhagic transformation in stroke means it could be highly dangerous to use for this specific application (Choi et al., 2011; Downs et al., 2015). Finally, the use of nanoparticles as a carrier method to enhance BBB penetrance of peptides is an exciting addition to therapeutic research that could revolutionize CNS drug delivery including OXT or an OXTR agonist (Zaman et al., 2018; Oppong-Damoah et al., 2019). While further investigation is needed, there is hope this can provide an effective, noninvasive, and safe option for OXTR modulation.

An additional challenge in OXTR targeting is that these receptors share around 40–50% homology with vasopressin receptors (Gimpl and Fahrenholz, 2001); moreover, these receptors can heterodimerize with each other (Terrillon et al., 2003). OXT and vasopressin differ at only two of their nine residues (Richter, 1983; Ivell and Richter, 1984). Vasopressin is also a partial agonist at OXTRs with only two residues in the binding pocket conferring a higher sensitivity to OXT (Chini et al., 1996). OXT can also act as a partial agonist at vasopressin receptors due to these similar binding sites (Mouillac et al., 1995). An excellent review of this issue and other considerations like bivalent agonists and G protein-specific ligands provides more detailed insight (Chini and Manning, 2007). More recently, truncated versions of OXT peptides have shown potency as agonists without off-target and dangerous V1a receptor agonism (Kablaoui et al., 2018). Surprisingly, the reduced molecular weight did not lead to an increase in permeability for the small cyclic analog (Kablaoui et al., 2018). These analogs then are a potential breakthrough for specific targeting of the OXTR in the periphery but does not solve the problem of BBB permeability for modulating CNS OXTRs.

The half-life of OXT has been studied extensively, but all show relatively short periods of action. In blood and plasma the half-life of OXT is only 4–5 min and in pregnant women even lower at 2–3 min (Leng and Sabatier, 2016). The half-life of OXT was reported to be higher in CSF or after an intracerebral injection reported at around 20 min (Mens et al., 1983). Regardless, the short window of action is likely not desirable for a drug meant to create long term behavioral modifications. One suggestion has been to use non-peptide agonists or antagonists for the receptor. Non-peptide agonists and antagonists can have a longer half-life as endogenous peptidases do not recognize such molecules (Evans et al., 1992). Many of the non-peptide OXT analogs tested to date have shown good to strong efficacy and affinity profiles (Hicks et al., 2012, 2014). However, there are currently no selective non-peptide OXTR agonists in clinical trials, although the WAY 267 464 agonist is available for basic research (Ring et al., 2010). Since the recent discovery that methylated WAY 267 464 becomes an antagonist at the OXTR instead of its typical agonistic effect, this has opened a possibility to further predictive models for specific nonpeptide agonists and antagonists (Jorgensen et al., 2016; Uba et al., 2020). Synthetic OXT peptides such as pitocin or syntocinon behave very similarly to endogenous OXT, whereas peptide analogs such as carbetocin have shown a longer half-life than OXT and fared better clinically (Manning et al., 2012). Another alternative that can prolong the half-life of the peptide is lipidation. In fact, Cherepanov and coworkers have created several analogs of OXT with palmitoyl groups added to various residues (Cherepanov et al., 2017). These analogs were able to induce behavioral changes out to 24 h indicating longer half-lives, though the ability to induce intracellular calcium uncaging through the OXTR was much less than the endogenous peptide (Cherepanov et al., 2017). With respect to receptor antagonism, OXTR antibodies bound to the surface of liposomes have been tested, but only in targeting myometrium at this point (Hua, 2019; Hua and Vaughan, 2019).

In summary, the complex nature of OXTR biology, with diverse, context-dependent cellular processing, homology with the vasopressin receptors, and ubiquitous expression peripherally and centrally, present challenges to the implementation of promising treatments. Nevertheless, that should not discourage the continuing search for specific and safe options. Certainly, the multitude of potential conditions, including ischemic injury, that might benefit from targeting the OXTR, and the history of successfully targeting GPCRs for treatments in general, should only serve to inspire more promising strategies and results in the future.

## Conclusion

Oxytocin was an early forbearer in peptide hormone research. As such, its “receptive substance” as proposed by Langley’s receptor theory (Maehle et al., 2002; Maehle, 2009) has inspired scientific interest in the OXTR for decades. Within this review, we have examined the foremost research on the functions of the OXTR at the cellular level and the consequences of this GPCR at the organismal level for both humans and animals, particularly with respect to vascular health and cerebrovascular dysfunction including stroke. These findings are summarized in Table 1. As translational research has come into its own, the structural, mechanistic, and behavioral data arising from OXTR studies support the utility of targeting these receptors in preclinical studies of cerebrovascular insults. In particular, there is therapeutic rationale for targeting the OXTR in the treatment and management of ischemic stroke and, potentially, vascular dementia. Secondly, the significant and ongoing amount of basic research into OXTR function should provide optimism that understanding the mechanistic role of this receptor in health and disease will continue to refine therapeutic strategies for these disorders.

**TABLE 1:** Potential effects of oxytocin receptor activation in cerebrovascular and post-stroke environment.

Domain	Effect	Citation
Cellular differentiation	Induces cellular proliferation through phosphorylation of MAPK (ERK5).	Hansenne et al., 2005; Devost et al., 2008; Stary et al., 2019
Cellular proliferation	Aids in cellular proliferation through PKC activation of eEF2 by dephosphorylation, and Src dependent mechanisms.	Cassoni, 2006; Devost et al., 2008; Deing et al., 2013
Cellular migration	The PLC pathway activates Akt/PI3K to influence endothelial cell migration. Reduces the transendothelial cell migration of immune cells.	Cattaneo et al., 2009; Viero et al., 2010; Erkanli et al., 2013; Liu et al., 2017
Synaptic plasticity	Leads to upregulation of plasticity related proteins and neurotrophins.	Havranek et al., 2015; Bakos et al., 2018
Vasoactivity	Promotes vasodilation in small vessels, but might promote vasoconstriction in large vessels; dependent on endothelial versus smooth muscle contributions.	Altura and Altura, 1984; Uvnäs-Moberg, 1998; Thibonnier et al., 1999b
Glucose uptake	Encourages the uptake of glucose in most cell types.	Lee et al., 2008; Florian et al., 2010
Autonomic nervous system	Increases parasympathetic tone over sympathetic tone leading to decreased reactivity.	Ebner et al., 2005; Huber et al., 2005
Blood pressure	Lowers blood pressure.	Petersson et al., 1996; Light et al., 2005
Heart rate	Decreases heart rate and cardiac output through ANP, however, some pregnant women experience increases.	Yashpal et al., 1987; Gutkowska et al., 1997; Rabow et al., 2018
Antioxidant Protection	Suggested to support an anti-inflammatory phenotype, but it remains unclear whether the receptor or peptide is more responsible.	Szeto et al., 2008; Rashed et al., 2011; Deing et al., 2013; Honceriu et al., 2016; Polshekan et al., 2016; Wang et al., 2018
Inflammation	Pushes most inflammatory cells toward an anti-inflammatory phenotype, though a specific mechanism is unknown. Candidates include activity at NF-κβ and RAGE.	Jankowski et al., 2010; Metz et al., 2012; Yuan et al., 2016; Wang et al., 2018; Garrido-Urbani et al., 2018; An et al., 2019

## Author Contributions

EM researched and served as the principle author of the review. SC served as the editor and secondary author of the review. Both authors contributed to the article and approved the submitted version.

## Conflict of Interest

The authors declare that the research was conducted in the absence of any commercial or financial relationships that could be construed as a potential conflict of interest.
